# Parents’ and children’s experiences of participating in a randomized controlled clinical trial: AIDIT-QS

**DOI:** 10.1080/17482631.2024.2408829

**Published:** 2024-10-03

**Authors:** Peter Sand, Emelie Kinberg, Frida Sundberg, Gun Forsander

**Affiliations:** a Department of Psychology, University of Gothenburg, Gothenburg, Sweden; b Region Västra Götaland, Department of Psychiatry for Affective Disorders, Sahlgrenska University Hospital, Gothenburg, Sweden; c Department of Pediatrics, Institute for Clinical Sciences, Sahlgrenska Academy, University of Gothenburg, Gothenburg, Sweden; d Department of Pediatrics, Region Västra Götaland, Sahlgrenska University Hospital, Queen Silvia Children’s Hospital, Gothenburg, Sweden

**Keywords:** Clinical trial, RCT, type 1 diabetes mellitus, children, parents, qualitative study, participation, expectation, attitudes

## Abstract

Participation in a paediatric, complex randomized controlled trial (RCT) might add to the family burden when a child is diagnosed with a severe disease. Although important, there are only a limited number of papers describing this aspect of research from the family point of view. This study explored parents’ and children’s experiences of participation in a research study shortly after the child had been diagnosed with type 1 diabetes. Sixteen parents (nine mothers, seven fathers) and nine children were interviewed by an independent researcher about their inducement, the decision-making process within the family which led to their participation, and their experience of having done so. The result showed that the parents wanted to contribute to improve treatment for children with diabetes in general but also specifically for their own child. Older children were more involved in the decision making than the younger children. Study information needs to be communicated clearly and effectively since decision-making based on information of a clinical trial directly after the child’s diabetes onset proved difficult. Being randomized to the intervention group in this specific study was considered somewhat burdensome. However, parental participants in both intervention and control group claimed that they would recommend participation in research studies to other parents in a similar situation, and so did the children. There was no difference between the mothers’ and fathers’ experiences.

## Implications

- Parents’ expectations: A predominant driving force for the parents was the expectation that the study outcome could lead to something good for both their own child and other children with type 1 diabetes.
- Children’s perspective on participation: Older children appreciated being involved in the decision-making process and valued their role in potentially helping others with diabetes. However, younger children were less involved and often relied on their parents for decision-making.
- Personal benefits: Both children and parents considered it important to gain something for themselves; by participating, they could benefit from more advanced technology and more rigorous follow-ups.
- Importance of control group: It was important for the families’ motivation for completing the study that the researchers conveyed that the control group was as important for the outcome of the study as the intervention group.
- Future treatments: The parents felt that participation in the clinical trial could eventually lead to new treatments that could help their own child.
- Perceived safety: The fact that the clinical trial was considered well-planned and safe and implied no risk for the child made it easier to agree to participation.
- Effective communication: Since the onset of diabetes is emotionally stressful, and diabetes treatment itself is demanding, effective communication about the content of such a clinical trial is necessary, otherwise families may not understand what they are agreeing to.
- Burden on the intervention group: This clinical trial was somewhat burdensome for the intervention group to participate in; nevertheless, all of the families remained committed to their reasons for participating and completed the study

## Background

Participation in clinical trials, particularly randomized controlled trials (RCTs), is crucial for advancing medical research and improving patient outcomes (Tunis et al., 2003). Understanding families’ experiences and motivations for participation is essential for enhancing recruitment strategies and the quality of RCTs (Shilling et al., 2011). Despite extensive research on individual motivations, there remains a notable gap in understanding the collective decision-making process within families as a whole, especially in challenging RCT settings (Hissink Muller et al., 2018; Johnson et al., 2007, 2009; Pagano-Therrien & Sullivan Bolyai, 2017; Shilling et al., 2011; Snethen et al., 2006). This gap highlights the need to investigate how family dynamics and individual perspectives influence participation decisions, particularly in paediatric healthcare interventions.

This study focuses on families’ experiences of participating in an RCT for children with type 1 diabetes. This is a challenging RCT setting, as the patients are children, and it involves a chronic disease that requires rigorous and lifelong management (Delamater et al., 2018). The treatment is demanding for the entire family and requires adjustments to their daily lives. It also has various psychological and emotional consequences, and the onset of the disease often being a shock to family members (Delamater et al., 2018; Streisand et al., 2008; Sand et al., 2018; Whittemore et al., 2012). Given these challenges, it is of valuable to investigate the experiences of the families who decide to participate in a RCT shortly after the onset of type 1 diabetes, to understand how it influenced their decision-making.

Previous studies on the experiences of participating in RCTs have highlighted related to the randomization process. A systematic review has shown that families often prefer to be randomized to the treatment group, which can negatively affect their willingness to participate in paediatric trials (Beasant et al., 2019). Similarly, in a study on psychological support for families of patients with type 1 diabetes, parents were more satisfied if they had been randomized to the treatment group (Sand, 2015). Another important aspect is the difference within the family, where one study indicated that parents’ and children’s motivation to participate in RCTs may differ. However, few studies have compared older children’s views with their parents’ preferences (Midgley et al., 2016).

Various motives for participation in RCTs have been described in previous studies, one of the most common being altruism, reported by both by parents and children (Barned et al., 2018; Drury et al., 2021; Glogowska et al., 2001; Midgley et al., 2016, Wulf et al., 2012). Another commonly stated reason is the desire to contribute to healthcare and research (Saarijärvi et al., 2020; Woolfall 2013).

The reported adverse experiences of participation in RCTs have been that it can be time-consuming and the feeling obligated to take part (Barned et al., 2018). Additionally, previous RCT participants have noted difficulties understanding the study’s content, the randomization procedure and what they actually consented to (Midgley et al., 2016; O’Keeffe et al., 2020).

### Research agenda

The first part of the RCT, Azithromycin Insulin Diet Intervention Trial (AIDIT), has been completed at the Queen Silvia Children’s Hospital, Sahlgrenska University Hospital, Gothenburg, Sweden in collaboration with the University of Gothenburg and Uppsala University. The aim was to induce beta cell rest and investigate whether the drug azithromycin, administered three times per week, extra dietician advice and support, in addition to repeated intensive insulin therapy by initially 72-hour i.v. and insulin pulses at seven occasions every 6–8 hours s.c. could help preserving insulin secretion in children who have recently been diagnosed with type 1 diabetes. The study aimed to recruit 50–60 children, aged from 6.0 to 15.99 years. The children participating in the RCT were randomized to treatment as usual (TAU) or to TAU supplemented with adjunctive therapy according to the AIDIT protocol. Both groups were offered the same number of follow-up visits during the first year. Within the first week after diagnosis, all children received a sensor augmented insulin pump and all families were offered contact with a study diabetologist or nurse at any time during the study year. The children were stratified according to age: 6–9.99, 10–15.99 years of age in order to take puberty in consideration. Due to the nature of the AIDIT protocol it could not be blinded to neither the participant or the investigator whether the participant was participating in the intervention or control group. After 12 months, the control and intervention groups were compared based on insulin secretion ability using a mixed meal tolerance test (MMTT) and MRI examinations of the pancreas, which were also conducted at diagnosis. The detailed study protocol and the metabolic outcomes after one year are described elsewhere (ClinicalTrials.govIdentifier: NCT03682640).

Only the older children (>10 y) were invited to participate in the qualitative part of AIDIT. Decision-making in clinical trials involving children can vary significantly based on the child’s age and developmental stage. Younger children must rely heavily on their parents or guardians to make health-related decisions, while older children and adolescents may have more input and desire to be involved in the decision-making process (Katz et al., 2016). This developmental perspective is crucial in understanding family dynamics and the motivations behind participation in clinical trials. In the context of the AIDIT study, children aged 10 years and above were involved in the qualitative interviews to capture their perspectives, acknowledging that their cognitive and emotional maturity allows for a more nuanced understanding of the trial and its implications.

## Aim

The aim of this part of the AIDIT study was to explore parents’ and children’s experiences of participating in an RCT immediately after the onset of type 1 diabetes and to evaluate how these experiences were perceived 12 months later.

## Methods

### Participants

The informants in this qualitative interview study were the first children and parents who had participated in and completed the RCT of the AIDIT-protocol. In total, sixteen parents (nine mothers and seven fathers) and nine children, mean age 12.4 years (range 10–15 years) participated. The sample included eight boys and one girl. Fifteen families were recruited, seven families from the control group and eight families from the intervention group. Four out of nine interviewed children had been randomized to the intervention group. Three approached families, all belonging to the control group, declined to participate in the qualitative study. However, no families from the intervention group declined participation. The mean of the HbA1c of the total group after completion of the RCT, 12 months after diabetes diagnosis, was 43 mmol/mol (6.1%).

### Procedure

Written and oral information on this added qualitative part of the AIDIT study was provided to both parents and to children above the age of ten years after the RCT was finished, following the guidelines outlined in the Consolidated Criteria for Reporting Qualitative Research (COREQ) (Tong et al., 2007). This comprehensive approach ensured that participants were adequately informed about the qualitative aspect of the study and could make an informed decision about participation. The information on this part of the study was communicated by the principal investigator of the study (GF) on the family’s visit to the clinic after the completion of the one-year RCT. After getting some days of afterthought, the informants were asked to access and sign consent forms. It was explained to the informants that participation was voluntary and that it was possible to end participation at any time if desired without affecting the child’s upcoming diabetes treatment. Mothers and fathers were interviewed separately, and the children were also interviewed individually.

### Data collection

This study had a qualitative design to explore the meaning and significance of parents’ and children’s experience of participating in an RCT immediately after the child’s diabetes onset. The interviews took place at the hospital-based outpatient clinic or, due to the COVID-19 situation, via online video meetings. The interviews lasted between 20–35 minutes, with no difference in duration between those conducted on site or those conducted via video meetings.

The interview guide was different for parents and children. Families who were in the intervention group had five additional questions, see the interview guides (Appendix 1and 2). These appendices outline the specific questions tailored to each group, highlighting both the similarities and differences in their perspectives on the study’s interventions and outcomes. The interview guide underwent a thorough review process, which included feedback and revisions by an experienced researcher (JS) specializing in thematic analysis, in accordance with the Consolidated Criteria for Reporting Qualitative Research (COREQ) guidelines (Tong et al., 2007). This ensured that the guide adequately captured the nuances of the research questions and provided a robust framework for conducting the interviews. The interview guides were semi-structured and covered the following areas: (1) the informant’s experience of participating in the RCT, (2) the decision processes within the family, (3) the motivations to participate in such a study, and (4) the informant’s experience of the randomization process. Additional questions for families in the intervention group covered how the informant experienced the study protocol elements and the possible treatment burden. All questions were open-ended to encourage the participants to express their own thoughts and experiences. Follow-up questions were used to a varying extent, depending on how much detail the participant supplied spontaneously. All interviews were audio-recorded and transcribed verbatim.

### Analysis

Inductive thematic analysis, as described by Braun and Clarke (2006), was used to analyse the interview data. The purpose of the inductive coding is to search for patterns, or themes, that are specific to each group’s experiences. Furthermore, the analysis was based on a critically realistic perspective, which means assuming that the participants’ subjective position affects their understanding and experiences of the phenomenon under study but at the same time, the feelings and experiences described by the informants are considered to be true and real (Braun & Clarke, 2006).

The interview transcripts for parents and children were treated separately in the analysis, as well as being separated according to whether the child was in the RCT intervention or control group, in line with the recommendations of the Consolidated Criteria for Reporting Qualitative Research (COREQ) guidelines (Tong et al., 2007). This approach allowed for a thorough exploration of the data while maintaining transparency and methodological rigour. The coding was carried out at a semantic level, as the focus of the study was on the experiences of the informants. Once all transcriptions were coded, the codes were organized and abstracted to enable thematization. One researcher (EK) generated 56 codes for the parents and 28 codes for the children. These codes were abstracted into four themes and seventeen sub-themes. Finally, the themes and sub-themes were revised and defined by two researchers (PS, EK).

Additionally, data saturation was achieved, indicating that a sufficient depth and breadth of experiences had been captured. This was determined during the last three interviews, where themes became repetitive, and no new insights emerged. This aligns with the principles of inductive thematic analysis, which seeks to identify patterns and themes until no new information is found.

#### Reflexivity

The initial idea of this interview study was raised by GF, who is the principal investigator of the AIDIT project and a paediatric diabetologist.

The author who conducted the data collection (PS) had earlier been working as a clinical psychologist at the same hospital-based outpatient clinic where the AIDIT clinical trial took place. Consequently, PS had a preunderstanding of the impact of type 1 diabetes for families, both emotionally and practically for their everyday life, as well as knowledge of diabetes self-care and the medical treatment procedures. This background ensured reflexivity and methodological transparency, as recommended by the COREQ guidelines (Tong et al., 2007).

Initial analysis of the interviews was carried out by EK, a psychologist with no prior experience in this context. EK received an introduction to type 1 diabetes in children and the RCT before conducting the coding. Themes and sub-themes emerged through collaborative work between EK and PS. The final refinement of themes and sub-themes was performed by PS.

## Results

Results from both parents’ and children’s interviews are presented with a focus on their motivations to participate in the RCT and how the decision to participate was made within the family. In addition, the overall experiences described by children and parents after participating in an RCT are also outlined. The findings consist of three main themes and six subthemes (Table I). The families who took part in the intervention group got additional questions about their experiences of the treatment itself. These results are presented separately under the third main theme.

**Table I.** Overview of the thematic structure.

Themes	Subthemes
Decision-making process	Motivations for participating
	Discussions within the family A mutual decision
Experiences of the clinical trial	Processing information in an overwhelming situation
	Experiences of the clinical trial design
Experiences of the intervention	Impact on everyday life
	The intervention as somewhat burdensome

### Decision-making process

The underlying reasons for participating in the clinical trial were partly altruistic and partly personal. All parents felt that it was important to involve the child in decision-making. The discussions that led to the families´ agreeing to participate in the study took place in two stages. First the parents discussed with each other and then they invited the child. All parents attempted to involve their child in the decision-making process, but older children had more influence on the final decision.

#### Motivations for participating in the RCT

There were three main motives for participating in the RCT. First, parents had the perception that their children would benefit by participation in the study, since they would have access to advanced technology as insulin pump and sensors already from the first week after diabetes diagnosis. All study participants, regardless of randomization group, were offered the most updated technical treatment which, by extension, could contribute to better health care for the child. One of the benefits for the child during participation was to receive medical technical tools faster, for example to receive pump treatment earlier than children in ordinary diabetes health care, as expressed by a mother of a boy: *“In part, it was this positive thing, that we got access to updated medical technical support as pump and sensor right away*.” Second, a strong reason for participating in the study was to be able to contribute to improving health care. Many parents also hoped for being randomized to the intervention group, with the expectation that the additional treatment for the intervention group would be effective:

Then I suppose we thought it would have been nice if he could be included in that study, I mean in the treatment group, if it could lead to something or yes … both contribute to research but mainly selfishly really, for his sake then and for our sake. (mother of a boy)

Third, a strong motive was the possibility of receiving more attention and help from the medical staff:

We have always been able to consult [a physician] or one of the nurses there about issues. It didn’t always have to do with the study and it felt like[son’s name] has been well taken care of thanks to this. (father of a boy)

One of the children expressed this as well: “*When I heard that I would get a lot of help out of it, ok then I thought I wanted to do it because I get more help and more answers*.”

#### The discussions within in the family

The decision to participate in the study was discussed among the parents before the child was involved. The discussion was often described as brief because many of the parents were largely in agreement on their reasons to participate. “I do not think we discussed that much (…) but we were pretty much in agreement that if this can help him, then we will do it.” *(mother of a boy)*. Most of the children could not recall the process that led to their making a decision to participate in the study. “*I don’t remember [about the discussion in the family]*” [*son´s name*]. Almost all parents explained how they first discussed the study between themselves before involving the child: “*We discussed*, [*partner’s name*] *and I, internally, by ourselves to find out where we stood together and then we also involved* [*son’s name*], *of course, so that we understood what he thought and felt*” *(father of a boy)*. Only one informant, the mother of a boy, was not in agreement with her partner at first: “*This sounds like a lot, a lot of effort so to say, so I was not that positive (…) it was actually* [*son’s name*], *himself and* [*partner’s name*] *who were the driving forces*.”

#### A mutual descision

All parents tried to involve the child in the decision-making and did so by explaining their own motivations for participation. The older children had more of an influence in deciding to participate in the study. Overall, the children had lower influence in the decision-making than their parents, and this was explained by their shock on receiving the diagnosis, the debilitation caused by diabetes, and their low age. For example, one mother of a boy said, “*I think he was pretty shocked and kind of absorbed in himself, so I don’t know how much he understood…*” Or one father of a girl, whose daughter was six years old at diabetes onset: “*Only that I guess we asked her and that I suppose she thought that it … but she probably didn’t really know what she was getting herself into*.” Nevertheless, many parents did mention that they wanted their child to take the final decision about participation but that, at the same time, they wondered whether their own wishes had influenced their child’s decision:

Then of course it’s hard to know how much we influenced [son’s name], if he had been allowed to choose completely independently … but I certainly think we influenced him in the direction of joining the study. (father of a boy)

All parents tried to involve the child in the decision and did so by explaining their own motivations for participation. They also explained what challenges the study would entail:

Both [partner’s name] and I, right from the start, were very set on joining so to say, but we needed to explain to [son’s name], what it meant, that we perhaps had to visit the hospital more often and that they would do tests and you will be x-rayed, and whatever it might be. (father of a boy)

### Experiences of the clinical trial

Since the onset of diabetes is emotionally stressful, and diabetes treatment itself is demanding, all parents reflected on the fact that the study had been presented early after onset. Interestingly enough, all families were satisfied with how the outcome of the randomization turned out.

#### Information processing in an overwhelming situation

All the parents reported that the information about the ongoing RCT came at an early stage after their child’s diabetes onset. It was hard for them to process the specific information about the clinical trial and separate it from the general information they were given about diabetes as such. In the interviews, they expressed different opinions about the initial information. Some parents thought it was understandable in every detail, while others thought it had been difficult to understand and interpret the information. For most parents, it was difficult to absorb the initial information about the RCT at the moment it was presented by the clinical staff, as several parents were affected emotionally at that time. One father described his state of shock:

In retrospect, we’ve probably reflected that yes, we didn’t know what we were saying yes to because it was very difficult to absorb that information as well as all the other information you get. (father of a boy)

Some parents subsequently understood that they had initially interpreted the information in a way that did not correspond to the actual intention of the study:

It has to do with how you interpret… (…) maybe I was probably more believing that it would lead to somewhere far in the future to a more cure, but this is just to slow it [the diabetes] down, it has become clear to me more and more in the course of time. (father of a girl)

#### Experiences of the clinical trial design

One aspect which emerged from the parents’ interviews was that they had experienced the study to be safe and genuine. *“It felt fantastic to get help(…) it feels incredibly safe to be part of a professional team.” (father of a girl)*. For them, this meant that it did not involve any risks or side effects. There was also a general trust and an awareness about scientific studies among many of the parents. Above all, most parents thought it was logical that they were asked to participate in the study at such an early stage: “*But it’s quite obvious they want the children at an early stage (…) so I didn’t experience it as anything strange*” *(father of a girl).*

Similar thoughts were expressed in different ways: “*I understand that you need to involve newly diagnosed cases in order for it to be applicable in the study*” *(father of a boy).*

Almost all parents from both the intervention group and the control group expressed that, in hindsight, it would have been worse for them to be in the other group. Parents also experienced the randomization as a standard procedure. Only one of the parents felt that the result of the randomization had influenced his act of participating in the RCT. While recognizing that being in the intervention group demanded more time and energy of the families, the parents in this group pointed out various benefits of ending up in this group. The first one was the opportunity for their child to receive effective interventions:

In the first stage, I also realized that much more will be required because he ended up in the intervention group and that we would have a longer hospital stay the very next week … but also positive that he would receive this treatment. (mother of a boy)

The children had similar reactions to their parents. “*And so I was very happy when I got it … [the intervention group]*” *(girl)*.

In the control group, parents talked about how not ending up in the intervention group had meant disappointment at first, but they now would not have preferred it because it entailed more time consuming visits at the hospital: “*We were a little disappointed at first, that he did not end up in the treatment group, but in retrospect I am happy about that, because I still think there were a lot of visits, extra visits*” *(mother of a boy)*.

The father of one child was an exception from all other informants, still expressing a little disappointment one year later about not ending up in the intervention group: “*Maybe we would have been a little more motivated [in the intervention group] … ”(father of a boy)*.

### Experiences of the intervention

The families reported both positive and negative experiences of participating in the study. On one hand it was burdensome for the intervention group to have to spend more time at the hospital and for the children to take antibiotics and more blood samples. On the other hand the families experienced a larger support in the diabetes care and an increased knowledge of diabetes.

#### The intervention as somewhat burdensome

The study protocol required regular times to be spent in the hospital. Most parents stressed that participation in the intervention group meant that there was less time for work and school. However, the parents considered it both justified and understandable that they were forced to be on site for the intervention itself.

Yes, demanding … it has been that you have had to take time off, but I have felt that like, it is for[son’s name] sake and for the sake of other diabetes children so for me it has not felt like it has been demanding in any way but I have done it gladly. (father of a boy)

There were also participants who considered the long days in the hospital to be monotonous, which was perceived as emotionally and mentally draining. *“It means extremely long days (…) there’s a lot of waiting, mentally, you don’t do much on a day like that, but mentally you’re completely drained when you get home” (father of a boy)*. A similar experience was presented by one of the children: “*It’s been a bit hard when I’ve been there during the day, that is, all day…” (boy)*.

Some parents pointed out that part of the intervention, taking a drug orally, affected their child’s well-being. One of the children said that the most stressful thing about participating in the study was taking medication every day. *“Taking medicine every day* [*about what’s been the hardest*].*”(boy)*.

The frequent need for laboratory samples was another feature in the study that had an effect on the child’s mood. “*He’s not really into needles*…” *(father of a boy)*. One child regarded participating in the study as burdensome. It was a difficult task for the whole family and something they obviously had discussed. “*It was much harder than we thought*.” *(boy)*.

Most of the parents in the intervention group commented on the insulin pulses. They considered these to be the most demanding part of the protocol. Some parents had difficulties in understanding the purpose of the pulses. Another aspect of this supplementary treatment was the insulin adjustments that had to be made after the insulin pulses. One mother said:

What has been most demanding is that after these insulin pulse treatments, I had adjusted his entire insulin needs, at least after some of them, so we have had to continue at home after such a treatment to find the right level again. (mother of a boy)

#### Impact on everyday life

Planning for and collecting antibiotics at the pharmacy was something the parents in the intervention group had to adapt to. It required more practical planning, such as trips to the pharmacy and remembering to go there. One father also highlighted that this extra task was something that parents needed to spend more time planning, but nevertheless felt that it had worked out relatively well.

He had to take the liquid antibiotic so (…) it was an extra factor that you wouldn’t have had otherwise, and[son’s name] probably hasn’t been really keen on it, but it still worked, it hasn’t been a big issue or anything, but of course, it takes that extra bit of time every week, you have to remember it and so on. (father of a boy)

Following the dietary advice was another extra task for participants in the intervention group only. This part of the protocol was not experienced as being not very demanding. “*It [the greatest burden] was probably this food diary (…) it ran over quite a few days (…) I can’t say it was really stressful really*” (*father of a girl)*. The intervention had an impact on everyday life, and the parents had to continuously work on the child’s and their own motivation. One parent considered that the technical advances offered during the study had helped him to maintain his own motivation. “*These techniques and statistics make you very committed and involved*” *(father of a boy)*.

Explaining to the child about the importance of following the study protocol was at times a challenge for the parents. Some children were concerned about missing school, and, in such cases, their parents had to repeatedly motivate them to continue with the RCT. “*So he doesn’t want to be away [from school] and so I have reinforced it all when we are coming here, talked about how important the study is (…) that we have to find a cure and help the research*” *(father of a boy)*.

One of the children expressed something similar: “*I thought that if it can help someone to feel better or whatever you say … or help someone else and it might work for me too maybe, then I thought it would be fun to contribute*” *(girl)*.

## Discussion

Little is known about the perspectives and motives of families who decide to participate in RCTs of treatment protocols where the child is the primary patient. This study aimed to address this gap by exploring the experiences of both parents and children participating in an RCT of the AIDIT treatment protocol for children with type 1 diabetes.

The decision-making process within these families was shaped by various motivations and how they discussed their potential participation. The main motivation for both children and parents was that it could lead to something good for the child and, by extension, for other children as well. These results are in line with previous research, in which altruistic motives for participating in studies have often been highlighted (Midgley et al., 2016). Both parents and children in the study were motivated by the prospect of accessing the latest technology, specifically an insulin pump and continuous blood glucose monitoring device, which were offered to both the intervention and control groups within one week after diabetes diagnosis. They also expected that participating in the study would lead to closer follow-up and an increase in their knowledge about diabetes. These reasons, which are linked to personal benefit for the child, are consistent with findings from a previous study (Tromp & van de Vathorst, 2019). Given these motivations, the term *conditional altruism* (McCann et al., 2010) might be applicable to this study. The concept of *altruism* has been the subject of some debate, and McCann et al. (2010) elaborated on it in a slightly different way from the everyday understanding of “helping others” to what they termed “*conditional altruism*”, where personal benefit is also considered when deciding whether to participate in an RCT.

The conditions for discussions within families varied based on the child’s age and health of the child. According to the parents, younger children had difficulties understanding what participation involved, while older children (aged ten and above) played a more active role in decision-making, collaborating with their parents to reach a consensus. Additionally, some parents reported that their child could not participate in the discussions about participation since they were psychologically affected by the onset of the illness. As a result, some parents acted as primary decision-makers, particularly for younger children, filtering information about research participation, especially when the severity of the illness was a factor. This strategy of parental filtering and decision-making is consistent with findings from Snethen et al. (2006), which highlighted similar dynamics in families dealing with severe illnesses.

Receiving information about the clinical study within a few days after a diabetes diagnosis was not without difficulties. Parents noted that they themselves had suffered emotionally because of the situation, which made it difficult for many to fully understand what they had consented to when they accepted the invitation to participate in the study. Research literature has well documented that the onset of the child’s diabetes often has far-reaching psychological consequences for parents, children, and sometimes siblings (Delamater et al., 2018; Sand et al., 2018; Streisand et al., 2008; Whittemore et al., 2012). The complexities involved in processing information related to clinical trials have been highlighted in other studies and are an area that needs constant evaluation and improvement (O’Keeffe et al., 2020; Wulf et al., 2012).

Regarding the experiences of the trial and its design, many participants initially hoped that the randomization process would place them in the intervention group. They believed this would ensure the child received a treatment that could potentially contribute to better somatic health. However, parents also viewed the randomization procedure as logical and accepted that it needed to occur at early. With one exception, families in both the control and intervention groups were satisfied with the outcome of the randomization. Additionally, parents felt that participation in either arm of the study was a valuable contribution. This reported satisfaction with the randomization contrasts with another study by Beasant et al. (2019). The parents’ experiences of the trial were also influenced by their high confidence in research in general, and particularly in the diabetes clinic running the RCT. They perceived the research as well-planned and felt it was free from risks. This confidence also positively affected the parents’ motivation to participate, with some parents assuming that their high degree of motivation likely influenced the child’s position.

Regarding experiences with the specific interventions of the AIDIT treatment protocol, participation in the intervention group was associated with certain challenges and strains. The children sometimes found the needle pricks painful and they experienced discomfort when taking medication. Most of the parents and children also highlighted their experience that spending long days in the hospital was monotonous. Another aspect of being in the intervention group was, from the child’s perspective, losing time for school and leisure, and from the parent’s perspective, it led to reduced working hours. Previous studies have highlighted the time commitment as one of the burdens on participants in clinical trials (Pagano-Therrien & Sullivan Bolyai, 2017; Barned et al., 2018; Greenberg et al., 2017). The parents sometimes needed to reinforce their own and the child’s level of motivation and had to emphasize to the child why it was important to follow the RCT protocol. The protocol eventually became part of the family’s everyday life.

### Limitations and strengths

One strength of this study is that the children and parents participating separately in the interview belonged to the same families, which allowed for an analysis of what the decision-making process within the family had been like from each perspective. The interviews also contribute to highlighting the older child’s perspective and voice when deciding to participate in a demanding RCT. Nonetheless, due to COVID, the interviews were largely conducted via video meetings, and the quality of the interviews might have been affected by these circumstances.

Detailed demographic data, including parent and child race, ethnicity, education level, and other relevant characteristics, were not fully captured. This limitation affects the comprehensive understanding of participant backgrounds and should be considered when interpreting our findings.

Regarding the AIDIT study, one limitation is that the intervention could not be blinded due to its specific design, particularly the insulin pulses. Participants in the qualitative follow-up knew what was offered and their responses were based on this experience. Unlike interventions involving a placebo control where the randomization outcome can be concealed, our study’s design inherently made this impossible. Therefore, it is important to note that not all intervention studies are equivalent, and generalizing our findings to all types of interventions, especially those with potential side effects, is not feasible.

This qualitative study only included children and parents who had agreed to participate in the clinical trial, which means that the study cannot present reasons for not participating. The small sample size, especially the limited number of children who were interviewed, makes it difficult generalize the results.

## Conclusion

Our study offers valuable insights into the motivations and experiences of families participating in clinical trials for type 1 diabetes. By examining both parental and child perspectives, we have illuminated the complex dynamics influencing enrolment decisions and engagement in these trials. Future research should continue to explore ways to optimize communication strategies and support mechanisms for families facing the challenges of participating in clinical research, particularly in the context of paediatric chronic illnesses like type 1 diabetes.

## Appendix 1. Interview guide for parents

1. How did you experience the announcement that your child had diabetes?
2. How did you experience receiving information about the AIDIT study right at the onset of diabetes?
3. What made you want to be part of this kind of study?
4. What was the process up to your decision to agree to participate? What was the driving force behind your participation in the RCT study? Did you involve the child in this decision?
5. How did you experience the randomization procedure?
6. How did you experience the outcome of randomization? Did the result affect your motivation to participate?
7. How do you think you would have experienced it if your child had ended up in the other group?

***Additional questions for parents in the intervention group:***

8. a. How have you experienced the usual insulin treatment (CGM, pump)? (*parents and children*)

b. How have you experienced the study treatment (additional insulin pulses, antibiotics three times a week, diet/dietician support)?

9. What have you found demanding about participating in the study?

10. Have you ever considered withdrawing from the study? What made you consider it?

11. Now that you have been part of a study, do you feel that the information you received at the time of diagnosis is consistent with how the AIDIT study was conducted?

12. If you were to give advice to others in a similar situation, what would you say to them about participating in a study like this?

## Appendix 2. Interview guide for children

1. How did it feel to find out you had diabetes?
2. How did it feel to be asked to participate in a study so soon after you got diabetes?
3. What made you decide to be in this type of study?
4. How did you talk about accepting the study in your family?

What made you say yes to participating in the study?

1. Did you get to decide for yourself if you wanted to be in the study?
2. How did it feel to be drawn randomly for one treatment or the other?
3. How did you feel about ending up in the group you were in? Did this affect your own willingness to participate?
4. How do you think you would have felt if you had ended up in the other group?

***Additional questions for children in the intervention group:***

9. a. What do you think about the usual insulin treatment (CGM, pump)? (*parents and children*)

b. What did you think about the study treatment (additional insulin pulses, antibiotics three times a week, diet/dietician support)?

10. What would you say has been the hardest thing about participating in the study?

11. Have you ever considered leaving the study? What made you think about it?

12. Now that you have been part of a study, do you think that the information you received matches how the intervention was carried out?

13. If you were to give advice to other children in a similar situation, what would you say to them about participating in a study like this?
